# Au-Ag-Cu nano-alloys: tailoring of permittivity

**DOI:** 10.1038/srep25010

**Published:** 2016-04-27

**Authors:** Yoshikazu Hashimoto, Gediminas Seniutinas, Armandas Balčytis, Saulius Juodkazis, Yoshiaki Nishijima

**Affiliations:** 1 Department of Electrical and Computer Engineering, Graduate School of Engineering, Yokohama National University, 79-5 Tokiwadai, Hodogaya-ku, Yokohama, 240-8501, Japan; 2 Centre for Micro-Photonics, Faculty of Engineering and Industrial Sciences, Swinburne University of Technology, Hawthorn, VIC 3122, Australia; 3 Melbourne Centre for Nanofabrication (MCN), Australian National Fabrication Facility, Clayton, VIC 3168, Australia; 4 Institute of Physics, Center for Physical Sciences and Technology, 231 Savanoriu Avenue LT-02300 Vilnius, Lithuania; 5 Center for Nanotechnology, King Abdulaziz University, Jeddah 21589, Saudi Arabia

## Abstract

Precious metal alloys enables new possibilities to tailor materials for specific optical functions. Here we present a systematic study of the effects of a nanoscale alloying on the permittivity of Au-Ag-Cu metals at 38 different atomic mixing ratios. The permittivity was measured and analyzed numerically by applying the Drude model. X-ray diffraction (XRD) revealed the face centered cubic lattice of the alloys. Both, optical spectra and XRD results point towards an equivalent composition-dependent electron scattering behavior. Correlation between the fundamental structural parameters of alloys and the resulting optical properties is elucidated. Plasmonic properties of the Au-Ag-Cu alloy nanoparticles were investigated by numerical simulations. Guidelines for designing plasmonic response of nano- structures and their patterns are presented from the material science perspective.

Facile synthesis of novel materials are opening new frontiers in the key areas of applications, e.g., a recent advances of perovskite solar cells reaching 20% efficiency milestone has been reached just after several years of the basic research^1^,^2^. Optical properties of dielectrics, semiconductors and metals are classified for the tailored absorbance or refractive index over ultra violet to infrared (UV-IR) spectral range and are used to design required optical properties of the layered films^3^. Recently, it was demonstrated that amorphous glassy state of mono-atomic metals can be created^4^. This opens new prospectives in alloying and formation of glass states of metals and tailor their thermal and electrical conductivity by control of the structure at atomic and nanoscale levels.

Currently, alloy nano-materials are being given significant attention due to emergence of their novel catalytic, energy storage, and optical functionalities beyond those of pure metals. It has been demonstrated that solid solutions of Rh and Ag mixed at the atomic level can store hydrogen much like Pd - a functionality non-existent in pure forms of Rh and Ag^5^. Another development of great interest is that Pd-Ru alloy nano-crystals provide for CO oxidization catalysis superior to that of Rh^6^. Unexpectedly, the Cu-Ag alloy also catalyzes CO oxidation^7^ and also exhibits anti-bacterial activity^8^. These new functionalities are related to the electron binding and charge distribution of the alloy which alters their chemical behavior, mostly defined by the electronic properties at the surface^9^. Also, such new metal alloys are strongly desired in plasmonic applications for spectrally tailored optical response. Using an electromagnetic field enhancement, a more efficient photo-voltaic systems have been reported^10^,^11^,^12^. Highly-doped semiconductors are utilized as low-loss plasmonic materials, i.e., TiN which is complementary metal–oxide–semiconductor (CMOS)-compatible and can be integrated in plasmonic photonic circuits^13^,^14^,^15^. These novel materials advance the field of functional intermixed metals and semiconductors beyond the well-established shape memory alloys, widely used due to their mechanical properties in bio-medical implant and space applications^16^,^17^.

Group 11 metals Au, Ag and Cu due to their high conductivity, chemical stability, thermal conductivity as well as a low thermal expansion are among the most widely used materials in the field of electronics. Also, they are prevalent in most plasmonic applications at the visible light spectrum due to their high concentration of free electrons and the *d*^10^ electron configuration which favors polarizability. Until now, most of the research on plasmonics has been conducted using pure metals, and their permittivity has been rigorously measured by means of ellipsometric studies^18^,^19^,^20^; it is noteworthy, that the permittivity of pure metals is strongly dependent on the film quality^21^. However, recently, numerous investigations were launched into plasmonic applications of dielectrics, semiconductors as well as other kinds of metals at the electro magnetic (EM) frequencies where damping losses are significantly lower. As part of this push, the importance and demand for affordable alloy materials with tunable properties for plasmonic applications such as surface enhanced spectroscopy/scattering (SERS) and surface enhanced infrared absorption (SEIRA) has been growing^22^,^23^,^24^,^25^,^26^,^27^,^28^,^29^,^30^,^31^,^32^, spurred on by prospects in quantitative detection of analytes at sub-1 ppm levels in the increasingly important fields of environmental monitoring, indoor air quality control, bio-medical sensing as well as inspection of chemicals in food, water and agriculture.

The current lack of knowledge of the complex optical properties of alloy plasmonic materials calls for direct measurement of relative permittivities of alloys at different compositions and to compare these results with analytical predictions obtained using the effective media theory. In previous work, it was shown how unreliable an estimate of alloy permittivity based the effective media formalism could be, even when the permittivity values for the pure constituent metals are known with high certainty, and how it is imperative to measure permittivity experimentally^33^,^34^. Optical, electrical, and mechanical properties of alloys are strongly dependent on crystalline structure as experienced by an electron, which in turn is largely determined by alloying conditions and, therefore, a wide range of values could be obtained for the same set of alloying components^35^. As the effective medium approximations typically neglect possible variations in alloy structure, systematic investigation over the full range of mixing ratios (from 0 to 100% of constituents) is necessary to obtain meaningful guidelines for predictive design of new plasmonic materials.

Here, the first study on the experimentally obtained complex values of permittivity for the *d*^10^ metals (Au, Ag, and Cu) alloys, covering the whole intermixing range of possible stoichiometric variations, deposited by means of thermal evaporation, is presented. Analysis of the optical properties of the alloys was carried out within the Drude model framework. Furthermore, X-ray powder diffraction (XRD) measurements were carried out in order to reveal crystallinity and intermixing of the constituent metals, thereby exposing the influence structural effects have on the plasmonic properties of an alloy. Finite-difference time-domain (FDTD) numerical simulations, based on the experimentally defined alloy permittivities, were conducted for generic nanoparticle-on-a-substrate test structures and revealed useful guidelines for control over the relaxation time and spectral position of their localized plasmonic resonances.

## Methods

### Samples and optical measurements

**Figure 1:**
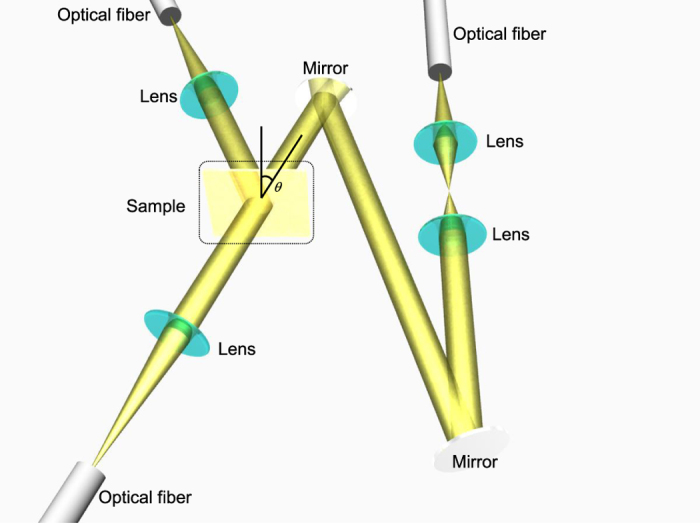
Layout of the optical setup used for simultaneous transmittance and reflectance spectral measurements. Sample was a metal nano-alloy film on a glass substrate.

The complex permittivity (the real and imaginary parts) of thin film alloy layers deposited on glass substrates was determined by measuring transmission and reflection spectra^34^. Thin alloy films were prepared using thermal evaporation onto a glass substrate (Matsunami No. 4, 0.35–0.45 mm in thickness). Chamber pressure was set below 6.0 × 10^−4^ Pa, evacuated using a turbo molecular pump. Samples were set onto a planetary stage and kept at room temperature during deposition with a sample-to-target distance of 40 cm. The Au, Ag, and Cu targets were placed onto dedicated tungsten crucibles with a 2 cm separation. Films with thicknesses of 20, 30 and 50 nm were deposited at a constant rate of 0.18 nm/min, controlled using a quartz micro-balance monitor. Alloy layers were formed by alternating the deposition of high purity Au, Ag and Cu metals with an alternating step of 1 nm for good intermixing. Schematics of the optical setup used for measurements of transmission/reflection spectra are shown in Fig. 1.

Fiber coupled halogen deuterium D_2_ lamp (L10290, Hamamatsu Photonics Co. Ltd.) was used as the UV-IR light source. Un-polarized light diffracting from the fiber was collimated into a 1 cm diameter beam which impinges onto the sample. The transmitted and reflected light was collected using a charge-coupled device (CCD) array detector (C10083CA-1050, Hamamatsu Photonics Co. Ltd.). The experimentally obtained spectra were analyzed using the FEDataAnalysis permittivity analysis software (Ohtsuka Electronics Co. Ltd.). The Drude-Lorenz model was fitted to the experimental data using least-squares method^36^. The free and bound electron derived permittivity is defined in the following form^37^:


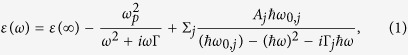


where the first two terms represent the Drude free electron model and the last term is the Lorentz contribution accounting for the bound electrons participating in interband transitions. Here *ε*(∞) represents permittivity at the high frequency limit (infinity), *ω*_*p*_ is the plasma frequency, Γ is the damping constant (=1/*τ*), *j* denotes the index of a Lorentz oscillator (a maximum index of *j* = 15 is assumed for higher accuracy of analysis), *A*_*j*_, *ω*_0,*j*_ and Γ_*j*_ are the amplitude, the resonant frequency and the damping constant of the given oscillator *j*, respectively, 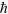 is the reduced Plank constant, *i* is the imaginary unit, and *ω* is the optical cyclic frequency. High fidelity numerical fits to experimentally measured refractive index data yield Drude parameters determined with high confidence bounds, with deviations less than 0.02%.

### Structural X-ray characterization

The crystallinity and grain size of the resulting alloys were investigated by means of X-ray powder diffraction (XRD) with RINT-2500 diffractometer (Rigaku Co.) and applying the 2*θ* – *θ* method. The angular resolution and scan rate were set to 0.02° and 0.5°/min, respectively. All alloy compositions at different mixing ratios of Au, Ag and Cu, were shown to be arranged in a face centered cubic (fcc) crystal lattice. Structural analysis was conducted by focusing on the diffraction peak from the (111) plane.

### FDTD calculations

The finite difference time domain (FDTD) numerical simulations of typical plasmonic nanostructures were performed using a commercially available software package FDTD Solutions (Lumerical Co.) and importing the experimentally determined complex permittivity values to define the material model. As a test structure an array of 200-nm-diameter disks of 50 nm thickness on a SiO_2_ substrate was modeled. This is a typical plasmonic structure often fabricated using electron beam lithography (EBL) and lift-off^30^,^31^,^32^,^38^,^39^. Periodicity of the disk pattern was set to 500 nm by using periodic boundary conditions along planes perpendicular to the substrate plane. The structures were illuminated from the substrate side using a broadband plane wave source and frequency-domain field monitors were set at the interface of metal nanostructures and substrate to obtain electric field distribution maps for determination of the local field enhancement factors. Field monitors were set above and below the structures for transmission and reflection spectral simulations. In order to obtain the optimal precision, a mesh override region with Δx = Δy = Δz = 2.5 nm maximum step size was set in the region surrounding the metallic structure.

## Results and Discussion

Simultaneous measurements of the transmission and reflectance of thin “Olympic” alloy films (Fig. 1) were carried out in order to determine their complex permittivity (*ε*_1_ + *iε*_2_), which in turn defines their optical properties.

### Optical permittivity and Drude parameters

**Figure 2:**
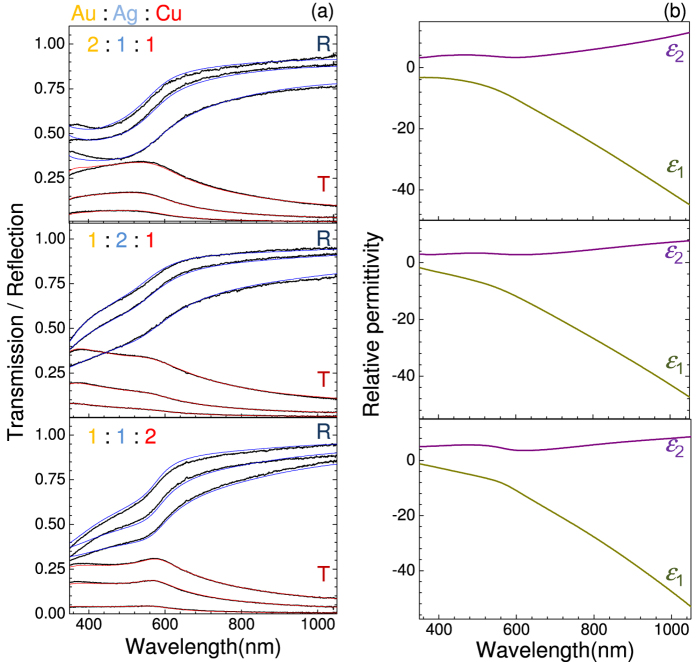
(**a**) Optical transmission, *T*, and reflection, *R*, spectra in the case of different atomic mixing ratios (Au:Ag:Cu) = (2:1:1), (1:2:1) and (1:1:2). Three sets of curves each represent layer thicknesses of 20, 30 and 50 nm, respectively. Red lines denote the best fit obtained using the Drude-Lorenz model (Eqn. 1). (**b**) Spectral dependence of the real, *ε*_1_, and imaginary, *ε*_2_, parts of the permittivity of corresponding alloys obtained from the Drude-Lorenz model analysis. All data analysis was done with *j* = 15 oscillators to achieve high fidelity *F* ≥ 0.9 fits.

Optical transmission and reflection spectra were measured for alloys with 38 different mixing ratios of Au, Ag and Cu, each deposited as films at thicknesses of 20, 30 and 50 nm. Examples of spectra taken at composition ratios of (Au, Ag, Cu) = (2:1:1), (1:2:1) and (1:1:2) are presented in Fig. 2a along with their best fit to the Drude-Lorenz model using least-squares method (see the experimental section with more details in the Supplement). The fitting parameter *F* = 1 − ∑_*i*_|Δ*Y*_*i*_|^2^/*N*_*data*_ was used to check for convergence. Here *N*_*data*_ denotes the experimental data points and Δ*Y*_*i*_ accounts for the difference between the experimental and simulated values. In this study, the theoretical model is considered convergent to the experimental results only when *F* ≥ 0.90. Oscillator number of *j* ≥ 12 is required for fitting experimental data for pure Au, Ag and Cu to be in agreement with permittivity as reported by Johnson and Christy^18^. For alloys it is expected to have a larger number of possible transitions and up to *j* = 15 oscillators were used to obtain a high fidelity fit. Thereby the optical permittivity (*ε*_1_ + *iε*_2_) in the UV-IR spectral range has been experimentally determined (Fig. 2b) for different mixing ratios of plasmonic *d*^10^ family metals with more details given in the Supplement. High fidelity fits which were consistently describing reflection and transmission of very different alloys collaborated validity of the used approach.

**Figure 3:**
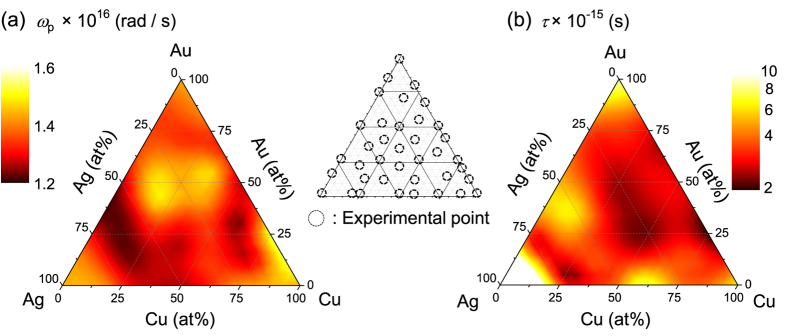
Interpolated ternary plots of experimentally obtained Drude parameters (**a**) plasma frequency *ω*_*p*_ and (**b**) relaxation constant *τ*. The Au:Ag alloy data were taken from the ref. 33. Middle inset indicates the atomic ratios at which experimental measurements were performed.

There was a tendency for Au rich alloys to exhibit large *F* values, most probably due to their higher chemical stability. Ag and Cu are susceptible to oxidization or/and sulfurization at room conditions. Permittivity of a given metal alloy is shown to be strongly affected by the nature of its stoichiometric constituents, i.e, enrichment in Ag leads to a decreased *ε*_2_ due to diminished damping of electronic oscillations typical for silver. Similarly, despite alloying, Au and Cu rich systems continue to exhibit characteristic interband absorption related spectral features in the longer wavelength region. Optical properties of metals are inextricably linked to the plasmonic response given rise by electrons oscillated due to the interaction with the EM-field of incident light. In order to quantitatively evaluate and compare plasmonic properties of Au:Ag:Cu alloys Drude parameters are extracted to account for the free electron contribution. Since in the shorter wavelength UV-visible (VIS) region an optical response is strongly affected by the interband transitions of bound electrons accounted for by the Lorenz oscillation terms, Drude parameters were extracted from experimental data at ≥650 nm wavelength. The resulting values of plasma frequency *ω*_*p*_ and the damping related relaxation constant *τ* for all 38 different Au:Ag:Cu alloy compositions are plotted in Fig. 3; the values for the Au:Ag system were determined previously^33^. Plasma frequency *ω*_*p*_ of the investigated alloys ranges from 1.2 to 1.6 × 10^16^ rad/s and directly corresponds to their free electron densities, which can be expressed as *ω*_*p*_ = (*Ne*^2^/*ε*_0_*m*)^1/2^, where *N* is the density of electrons, *e* is the elementary charge, *m* is the effective mass of an electron. Therefore, the observed spectral variations in different alloys can, to a large extent, be modeled as changes in *ω*_*p*_, e.g., an increase in the free carrier density results in a blue-shift of the plasmon resonance wavelength. For the Au, Ag, Cu alloy system under consideration, the middle of the area plot of Fig. 3a (delimited by the 75:50:50% lines for Au:Ag:Cu respectively) exhibits high plasma frequency values. Conversely, the other parts, especially ones rich in Ag are shown to have lower values. Since *τ* corresponds to the lifetime of the free electron oscillation, it directly affects the magnitude of plasmonic EM field enhancement. While, as expected, pure metals have large *τ* values, it is noteworthy that alloy systems around Ag (50–60%) Au (40–30%) mixed with ~15% Cu, as well as at Cu 60% Ag 40% seem to also show relatively decreased damping. Sufficiently narrow confidence intervals of 0.02% for the Drude parameters highlight the potential in plasmonic applications of such alloys. Plasmonic response of alloy nanoparticles are discussed next.

### Plasmonic properties of alloy materials

**Figure 4:**
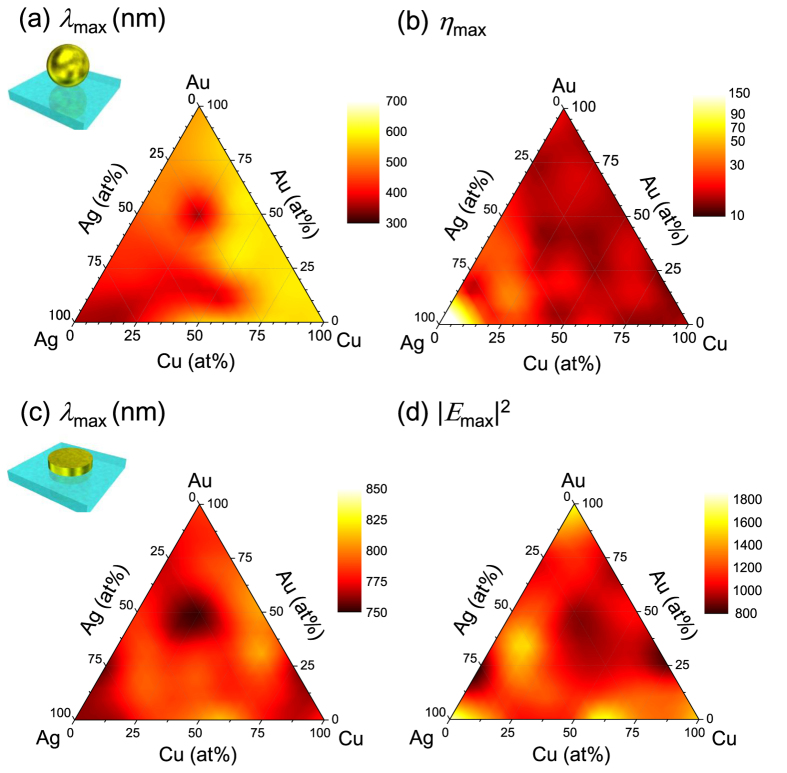
Summary of FDTD modeling and numerical calculations. (**a**) The maximum wavelength at which the strongest EM intensity was obtained for nano-spheres. (**b**) Maximum field intensity for the sphere. (**c**) Wavelength of the plasmon resonance for the nano-disk array. (**d**) Maximum |*E*|^2^ for the nano-disk array. The Au:Ag alloy data were taken from ref. 33.

For plasmonic applications of alloys it is of significant importance how plasmon resonance frequencies and EM field intensity values relate to their stoichiometric composition. For this purpose FDTD and numerical simulations of simple plasmonic structures were performed, using the experimentally determined complex permittivity values (*ε*_1_ + *iε*_2_) to approximate material properties of Au:Ag:Cu metal alloys. When conducting numerical analysis of the plasmonic resonance for a spherical metallic particle in the quasi-static approximation one can make use of analytical expression for EM field enhancement *η*^40^:


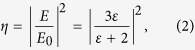


where *E*_0_ represents the electric field of the incident light and *E* is the localized electric field in the near-field of the plasmonic particle. The ternary plots of spherical particle resonant wavelengths and their corresponding maximum values of *η* for various alloy systems are presented in Fig. 4a,b, respectively. Equation 1 is only valid for a sphere, hence, FDTD calculations were carried for nano-discs (Fig. 4c,d). Using alloys, it is possible to tune the plasmonic resonance over the entire visible spectral range. Such flexibility is required in applications of color filters and photo-catalysts. A plasmon wavelength range from 350 to 650 nm was used to monitor plasmon resonance for the spheres of different elemental composition. Simulation results match previous experimental and numerical results^25^,^26^. This wavelength region is strongly affected by the interband transitions. In the case of nano-disc simulations, a wide range of plasmon resonances from 750 to 825 nm were obtained. The trend observed for the composition dependent resonant peak wavelength shift closely follows results obtained for the spherical structure, except for the Cu-rich nano-discs. This result indicates that the plasmon resonance of Cu spheres is red shifted due to the interband transition of Cu (the Lorenz oscillation). However, in the case of nano-discs, plasmon resonance is mainly defined by the Drude contribution and the difference between Au, Ag, Cu and their alloys become less significant, hence, the plasmon resonance range becomes narrower.

Furthermore, for several alloy systems the EM enhancement was shown to exceed that of pure Au and Cu. In the case of spheres, Au:Ag series with 15% to 5% of Au, Ag:Cu series with 20~25% of Cu, and with 40~60% of Ag in Au:Ag:Cu systems. Similarly high enhancements were observed for nano-discs in Ag rich compositions (Au 35%, Ag 52%, Cu 13% and Ag 40% Cu 60%). The overall EM enhancement trend is consistent with the relaxation constant *τ* of those alloys. For the higher *τ* values, electrons experience lower resistance (a high quality factor *Q* for the plasmonic resonance) which results in a strong EM field enhancement.

### X-ray crystallography

**Figure 5:**
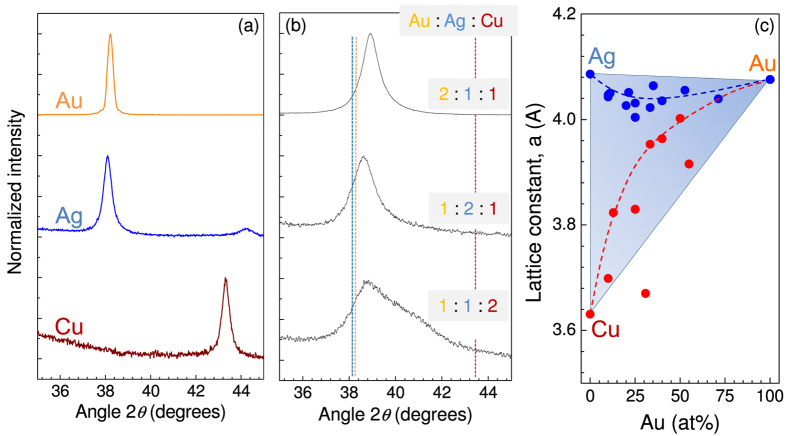
X-ray crystal diffractogram of pure Au, Ag and Cu metals (**a**), and their alloys at (2:1:1), (1:2:1) and (1:1:2) stoichiometric ratios (**b**). (**c**) Lattice constant, *a*, dependence on the Au concentration in the alloy system, obtained form the X-ray diffraction angle. Dashed lines in (**c**) are guides for the eye.

To gain further insight into the structural properties of Au-Ag-Cu alloys and to deduce their influence on the optical response crystallographic investigation by means of X-ray powder diffraction measurements has been performed (Fig. 5). In the case of pure metals, diffraction angles 2*θ* of around 38° for both Au and Ag, and 43.5° for Cu were obtained as expected. These values are directly related to their lattice constants a = 4.078 Å (Au), 4.086 Å (Ag), and 3.615 Å (Cu)^41^. Phase diagram for the metal alloys indicates that at all ratios of composition the Au-Ag system is arranged in a face centered cubic (fcc) lattice. Conversely, the Ag-Cu system has an fcc eutectic point at Ag_3_Cu_2_ at the eutectic temperature of 800 °C, however, provided splat cooling occurs, a quasi-stable solid solution state can be obtained in a wide range of compositions. The Au-Cu system exhibits a super lattice of Cu_3_Au, CuAu and CuAu_3_, where AuCu has a cubic lattice and the two remaining sub-lattices show the fcc arrangement.

In this study, we have focused exclusively on diffraction from the (111) face, since diffraction peak intensities from other planes are much weaker, therefore it would be exceedingly difficult to distinguish between the aforementioned cubic and fcc sub-lattices. A strong (111) signature indicates that the alloy has a fcc lattice. With the addition of Cu into the Au-Ag system, the diffraction peak associated with Au and Ag moved towards larger 2*θ* angles. However, at high concentrations of Cu, diffraction peaks become broader and apparently composed of two distinct peaks. This can be attributed to the solid solution formation during the physical vapor deposition step giving rise to heterogeneity in the metal alloy, which in turn is observed as two peaks corresponding to different lattice constants. Figure 5(c) shows how the lattice constant associated with observed diffraction peaks depends on the atomic ratio of Au in the alloy. As the system is enriched in Au the two peaks begin to shift closer to each other, converging towards the lattice constant of Au. From this result it can be deduced that Au containing alloys perfectly intermix, avoiding segregation into separate phases. Homogeneous crystal phase has lower electric dumping due to reduced grain boundaries and scattering centers. which favors larger EM field enhancements as discussed below. Surface enhanced Raman scattering (SERS) from such alloys with expected high EM-enhancement is an interesting topic for further investigations. Chemical attachment of different analytes on the surface of alloy with different neighboring atoms Ag, Au, or Cu is expected to be reflected in SERS chemical and EM-enhancement factors.

**Figure 6:**
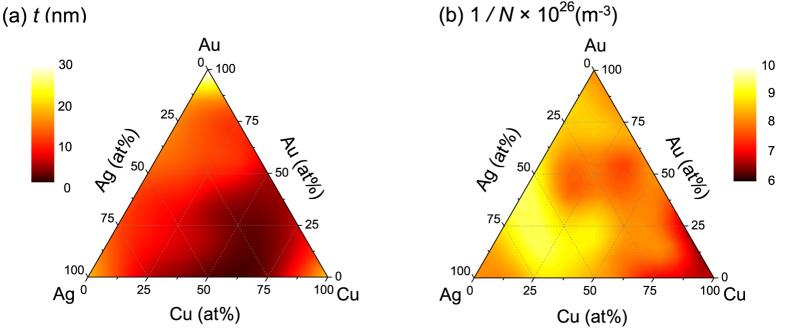
(**a**) Ternary plot of the size, *t*, of crystallites obtained from Scherrer’s analysis of XRD data (Eqn. 3). (**b**) relaxation time which is reciprocal to the density of scatterers, 1/*N*, of the alloys at varying stoichiometric compositions.

From the XRD measurement results it is also possible to retrieve the mean size of crystallites. According to the Scherrer’s equation, size of crystallites *t* can be calculated using the standard expression^42^:


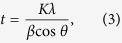


where *K* is the dimensionless shape factor in our case assumed to be 0.9, *λ* is the X-ray wavelength of 0.154 nm for the Cu-K*α* line used, *β* is the full width half maximum (FWHM) of the XRD peak in radians, and *θ* is the Bragg diffraction angle. The crystallite size values extracted from experimental data are plotted in Fig. 6a. The lattice constant of the fcc structure, *a*, and atom density, *n*_*a*_, were obtained based on the values of the diffraction angle *θ*.

Plasmon relaxation constant *τ* is the average duration between two scattering events which define the electron mean free path (mfp). Therefore, from the density of scatterers we can deduce *τ* according to the following equation:


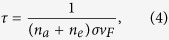


where *n*_*a*_ and *n*_*e*_ respectively are the densities of atoms and electrons, *σ* is the scattering cross-section, and *v*_*F*_ is the Fermi velocity of electrons. The *n*_*e*_ value, which defines the plasma frequency, can be obtained assuming that, as is typical for metals, one electron per atom is donated for the metallic bonding in the alloy. It is noteworthy, that other scattering mechanisms, e.g., by grain boundaries of nano-crystallites also contribute to the decrease of the mfp, i.e., cause a shorter relaxation time *τ*. The dependence of scatterer density 1/*N* = 1/(*n*_*a*_ + *n*_*e*_) on the alloy composition is plotted in Fig. 6b. It shows strong correlation with the ternary plots of *τ* (Fig. 3b) obtained from purely optical measurements. Compositional map of crystallite size *t* (Fig. 6a) can also be seen as representing the relaxation time component due to the grain boundary derived scattering *τ* ∝ *t*.

Mixing of metals in nanoparticles with different surface to volume ratios can bring new ordering of eutectic metal mixtures as predicted for the Ag_6_-Cu_4_ system, with prevalent formation of glassy mixtures with an icosahedral ordering around Cu atoms^43^. Alloy films prepared by evaporation as studied here could serve as a source material to make alloy nanoparticles using laser ablation via specific formation mechanisms exploring surface super-cooling/heating^44^ and fragmentation mechanisms^45^. Surface composition and ordering of alloy atoms is expected to bring new (photo) catalytic properties and surface functionalization scenarios for SERS sensing. Laser ablation of alloys using ultra-short laser pulses which also facilitates an ultra-fast thermal quenching could open a new availability of amorphous metals for SERS and photo-catalysis. This is an unexplored area of research since pure metals are forming crystals but not glasses.

## Conclusions

The optical permittivity (*ε*_1_ + *iε*_2_) of the plasmonic *d*^10^-metal Au, Ag and Cu alloy system was determined for the first time by means of a systematic experimental investigation over a large number (38 alloys) of intermixing ratios. The Drude parameters were extracted by numerical analysis of the optical transmission and reflection spectral measurements using a high fidelity fit. It is shown that the plasmon resonance wavelength can be tuned in a wide spectral range by simply changing the composition of the Au-Ag-Cu metal alloy, providing a way to engineer the plasma frequency *ω*_*p*_ of the system. It was confirmed that the plasmon resonance relaxation time, *τ*, which governs the EM field enhancement, is dependent on the crystallinity of the alloy. Strong correlation between *τ* values determined by means of XRD and optical measurements is shown. At certain alloy stoichiometries the EM field enhancement factor exceeds values observed for pure Au structures. Due to its resistance to oxidation and sulphurization at atmospheric conditions, the alloy system has strong potential for a variety of plasmonic applications including sensing, photo-catalysis, and solar energy harvesting. New chemical and EM-enhancements in SERS are expected on surfaces of these plasmonic metal alloys.

## Additional Information

**How to cite this article**: Hashimoto, Y. *et al.* Au-Ag-Cu nano-alloys: tailoring of permittivity. *Sci. Rep.* **6**, 25010; doi: 10.1038/srep25010 (2016).

## Supplementary Material

Supplementary Information
