# Supplementary material for: Au-Ag-Cu nano-alloys: tailoring of permittivity
Source: Sci Rep. 2016 Apr 27;6:25010. doi: 10.1038/srep25010 (PMC4846990; doi:10.1038/srep25010)
Supplement: Supplementary Information [file srep25010-s1.pdf]

Au-Ag-Cu nano-alloys: tailoring of permittivity

Yoshikazu Hashimoto[1], Gediminas Seniutinas[2,3], Armandas Balcytis[2,3],
Saulius Juodkazis[2,3,4], and Yoshiaki Nishijima[1,*]

1: Department of Electrical and Computer Engineering, Graduate School of Engineering, Yokohama National University, 79-5 Tokiwadai, Hodogaya-ku, Yokohama, 240-8501, Japan

2: Centre for Micro-Photonics, Faculty of Engineering and Industrial Sciences, Swinburne University of Technology, Hawthorn, VIC 3122, Australia

3: Melbourne Centre for Nanofabrication (MCN), Australian National Fabrication Facility, Clayton, VIC 3168, Australia

4: Center for Nanotechnology, King Abdulaziz University, Jeddah 21589, Saudi Arabia

SUPPLEMENTARY INFORMATION

Experimentally obtained all permittivity data

Figure Supplement shows permittivity spectra of pure metals and their binary and ternary alloys made in this study. All the data are plotted after the same analysis of experimental reflection and transmission measurements as shown in Figure 2. It is clearly discernable that real and imaginary parts of the alloys closely follow the same trend which signifies a good nanoscale alloying.

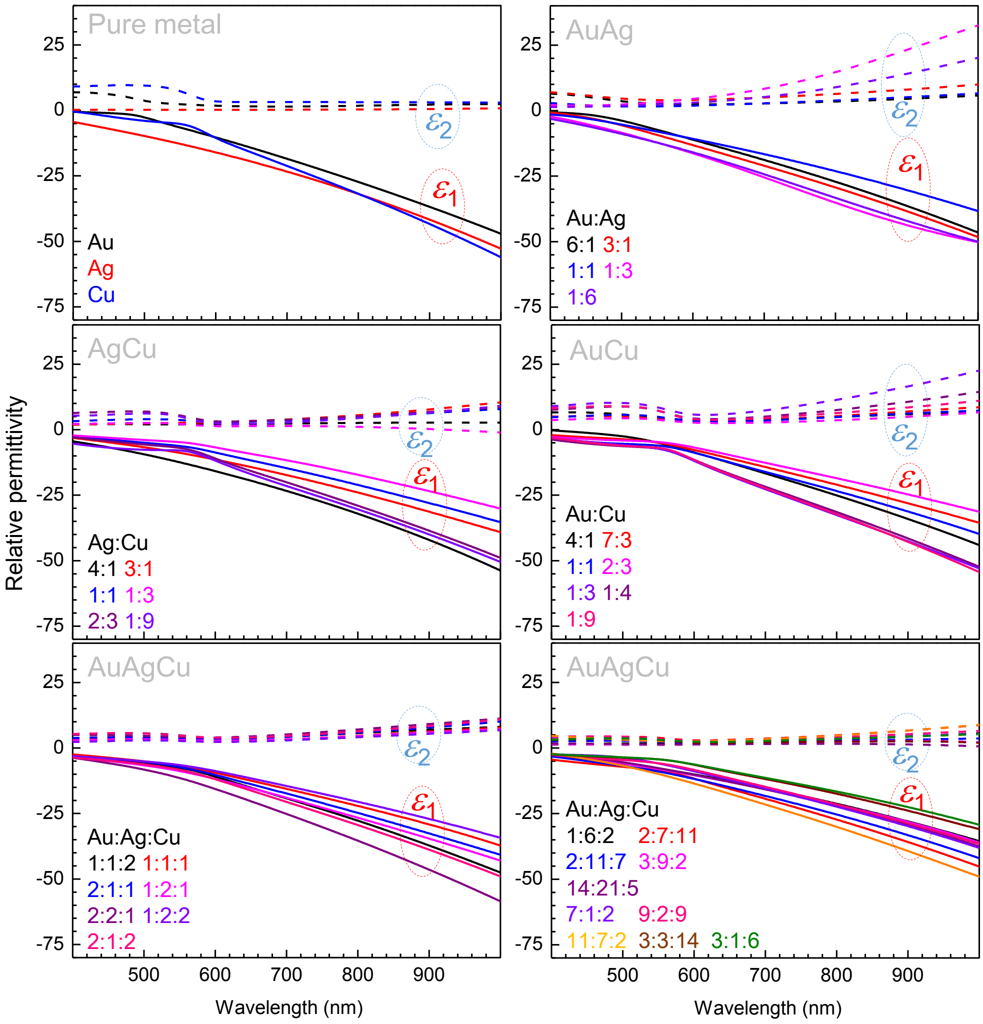

Figure Supplement. Spectra of the real, ε_1, and imaginary, ε_2, parts of the permittivity of pure metals, binary and ternary alloys at different mixing ratios (color coded) made by Drude-Lorentz analysis. All data analysis was done with j = 15 oscillators to achieve high fidelity $F > 0.9$ fits of the experimental reflection and transmission data (same as for Fig. 2 in the main text).
